# microRNA-4701-5p protects against interleukin-1β induced human chondrocyte CHON-001 cells injury via modulating HMGA1

**DOI:** 10.1186/s13018-022-03083-8

**Published:** 2022-04-22

**Authors:** Hui Zhang, Cheng Chen, Jie Song

**Affiliations:** 1 Department of Orthopedics, Huangshi Central Hospital, Edong Healthcare Group, Affiliated Hospital of Hubei Polytechnic University, Huangshi, 435000 China; 2 Department of Geriatrics, Huangshi Central Hospital, Edong Healthcare Group, Affiliated Hospital of Hubei Polytechnic University, No. 141 Tianjin Road, Huangshi, 435000 China

**Keywords:** Osteoarthritis, Chondrocytes, miRNA-4701-5p, HMGA1

## Abstract

**Background:**

miRNA-4701-5p has been reported to be a vital regulator in many diseases, including rheumatoid arthritis, and miRNA-4701-5p is evidenced to be participated in synovial invasion and joint destruction. In our report, we investigated the roles of miRNA-4701-5p in osteoarthritis (OA) and analyzed the molecular mechanism.

**Methods:**

Interleukin-1β (IL-1β) was applied for stimulating human chondrocyte CHON-001 cells to establish an OA injury model. mRNA levels and protein expression were measured using qRT-PCR and western blot assay, respectively. The proliferation ability and cytotoxicity of CHON-001 cells were checked using MTT assay and lactate dehydrogenase activity. The inflammation of chondrocytes was accessed by the secretion levels of interleukin-6 (IL-6), interleukin-8 (IL-8) and tumor necrosis factor (TNF)-α. The apoptosis of chondrocytes was determined by flow cytometry assay. Bioinformatics software Starbase v2.0 analyzed the functional binding sites between miRNA-4701-5p and HMGA1 and the interaction was further confirmed using dual luciferase reporter analysis. Results: miRNA-4701-5p was down-regulated in the IL-1β-stimulated chondrocytes and HMGA1 directly targeted miRNA-4701-5p. Up-regulation of miRNA-4701-5p could alleviate IL-1β-treated CHON-001 cells inflammation and apoptosis, and reversed the cell proliferation decrease and cytotoxicity increase after IL-1β treatment. Nevertheless, all the roles of miRNA-4701-5p overexpression in CHON-001 cells could be reversed by HMGA1 up-regulation.

**Conclusions:**

miRNA-4701-5p could alleviate the inflammatory injury of IL-1β-treated CHON-001 cells via down-regulating HMGA1, indicating that miRNA-4701-5p/HMGA1 is a promising therapeutic target for OA.

## Introduction

Osteoarthritis (OA), one of the most frequent joint disease in the world, is reported to be the leading cause of disability and has a great influence on people's physical and mental health, especially the elderly [1, 2]. Osteoarthritis is characterized by cartilage degradation, synovial inflammation, osteophyte formation and subchondral osteosclerosis [3, 4], in which the structure of articular cartilage is difficult to renew after damage, so articular cartilage damage is a vital pathological element resulting in osteoarthritis [5, 6]. Articular cartilage only depends on cartilage cells to maintain the extracellular matrix, and studies have shown that phenotypic changes in chondrocytes, such as cell hypertrophy and stromal calcification, could lead to cartilage degeneration in OA [3, 7, 8]. In late stage of OA, the chondrocytes were reduced, accompanied by emptying of joint space, which providing evidences indicate that chondrocytes death is a key trait in the progression of OA [9]. However, for a long time, the role of chondrocytes in cartilage degeneration of osteoarthritis has not been clearly clarified, in recent years, many researches indicated that chondrocytes could be used as a target for repairing joint degeneration [7, 10].

Increasing evidences have shown that non-coding RNAs, including microRNAs (miRNAs), long-chain non-coding RNAs (lncRNAs) and circular RNAs (circRNAs), were involved in various biological processes [11]. Mechanically, circRNAs and lncRNAs usually competitively sponge to miRNA, thereby alleviating the suppression of miRNA on its target genes and improving target genes expressions [12, 13]. Recent studies have shown that interactions between RNA and protein were involved in a variety of pathological processes of OA [14, 15]. Coutinho et al. revealed that 142 miRNAs and 2387 mRNAs were dysregulated in articular cartilage of OA patients, and suggesting that one miRNA can mediate the expressions of various genes, similarly, one gene can be regulated by many miRNAs [16]. Zhong et al. [17] suggested that miRNA-335-5p was memorably down-regulated in chondrocytes of OA patients, and it could reduce the inflammatory response by activating autophagy of chondrocytes. Skrzypa et al. [18] revealed that miRNA-146a-5p were highly expressed in the serum and cartilage of OA patients, and the expression level of miRNA-146a-5p in the serum could be used as a biomarker for the development of OA. Bi et al. [19] suggested that miRNA-4701-5p was memorably down-regulated in rheumatoid arthritis, and miRNA-4701-5p overexpression could inhibit synovial invasion and joint destruction, and there has been no report on the role of miRNA-4701-5p in OA yet.

High mobility group proteins (HMG proteins) are the most abundant group of chromatin proteins in eukaryotic cells after histones [20, 21]. The HMG protein family could be divided into three subunits, including HMGA, HMGB and HMGN. They are evidenced to be involved in chromatin structure and function as well as gene expression regulation [21–23]. HMGA1 has been confirmed to be related to a variety of cancers, and the expression of HMGA1 was significantly upregulated in various cancers [24, 25]. It has been reported that miRNA-26a down-regulates HMGA1 by targeting the 3'-UTR of HMGA1 and remarkably inhibits osteosarcoma cell lines migration and invasion [26]. And miRNA-142-3p has been reported could inhibit the progression of osteosarcoma cells via targeting HMGA1 [27]. Zheng et al. [28] indicated that miRNA-98-5p could prevent bone regeneration via downregulating HMGA2. Recent reports have suggested that HMGA1 was highly expressed in cartilage from patients with OA, and HMGA1 was proved to affect the proliferation and differentiation of chondrocytes [29, 30], which might be a pathogenesis factor of OA.

In our investigation, we analyzed the functions and relationship between miRNA-4701-5p and HMGA1 in an in vitro model of OA chondrocytes.

## Materials and methods

### Cell culture and treatment

The human chondrogenic cell line CHON-001 and HEK293T cells were bought from ATCC and maintained in DMEM (Gibco, CA, USA) supplemented with 10% FBS (vol/vol) (Gibco, cat no. 10100147), 100 μg/ml penicillin/streptomycin (Gibco, cat no. 15140148). The cells were cultured in a humidified environment at 37 °C and 5% CO_2_. The CHON-001 cells were exposed to indicated concentration (10 ng/ml) IL-1β (Merck, cat no. SRP6169) for 12 h to establish OA model.

### Cell proliferation ability

Cyquant MTT cell viability assay kit (Invitrogen, cat no. V13154) was applied for accessing CHON-001 cells proliferation ability. In short, cells were inoculated into 96 well plates with a density of 6 × 103 cells/well at 100 μl and cultured for 48 h. Then cell medium was replaced by fresh culture medium, and 10 μl MTT solution (12 mM) and 100 μl SDS-HCl solution were put in each well according to the instructions. Finally, the OD_570_ was measured using Varioskan™ LUX multimode microplate reader, Absorbance and Fluorescence intensity (Thermo, cat no. VL0000D0).

### Lactate dehydrogenase (LDH) quantification

LDH Cytotoxicity Assay Kit (Beyotime, cat no. C0017) was adopted for LDH quantification. CHON-001 cells were inoculated into 96 well plates at a density of 6 × 10^3^ cells/well in 100 μl medium until the confluence reached 70%. Then the culture medium was displaced by fresh medium and treated with indicated concentration (10 ng/ml) IL-1β and PBS was used as negative control, and cultured for 11 h. Then 10 μl LDH release reagent was added to each well and continue cultured 1 h. The plates were centrifuged, and LDH test reagent was added into supernatant at room temperature without light for 30 min. At last, we determined the OD _490_ using a microplate reader (BioRad, USA).

### Flow cytometry assay

CHON-001 cells apoptosis was determined using Annexin V-FITC Apoptosis Detection Kit (Beyotime, cat no. C1062M). The processed CHON-001 cells were obtained and washed by pre-cold PBS buffer and 5 × 10^5^  cells were resuspended with 195 μl Annexin V-FITC binding buffer. Then 5 μl Annexin V-FITC and 10 μl PI staining solution (1 mM) were put in and hatched for 20 min without light. Then FACSCalibur flow cytometry (BD Biosciences, USA) was applied to analyze cell apoptosis.

### Western blot analysis

CHON-001 cells were harvested using RIPA lysis buffer (Beyotime, cat no. P0013B). The cell fragments were removed by high-speed centrifugation and the supernatant containing total protein was preserved for western blot analysis. The supernatant containing 20 μg protein was split in 12% SDS-PAGE, and then transferred onto PVDF membranes by XCell SureLock Mini-Cell electrophoresis system (Thermo). The membranes were cultured in 5% skim milk in PBS buffer and then cultivated with specific primary antibodies (1:10,000 for anti-HMGA1, Abcam, cat no. ab129153; 1:500 for anti-GAPDH, Abcam, cat no. ab8245). Then the membranes were incubated with secondary antibodies (1:5000, cat. no. ab125919). The enhanced chemiluminescence (Cytiva) was used to detect the immune response bands.

### Enzyme-linked immunosorbent assay (ELISA)

The release of inflammatory cytokines, including IL-6, IL-8 and TNF-α were detected using ELISA. The detection kits were purchased from Beyotime (Human IL-6 ELISA Kit, cat no. PI330; Human IL-8 ELISA Kit, cat no. PI640; Human TNF-α ELISA Kit, cat no. PT518). The cell supernatant was collected by centrifugation and 100 μl supernatant was added to specific plate providing by the kit and incubated for 2 h at room temperature. Then the plate was washed, incubated with 100 μl biotinylated antibody for 1 h, and mixed with 100 μl horseradish peroxidase labeled streptavidin for 20 min in dark at room temperature. Then, 100 μl TMB development solution was put in and incubated for 10 min in dark. At last, we added 50 μl stop solution and analyzed the results at OD_450_.

### Quantitative reverse transcription PCR (qRT-PCR)

The expression of miRNA-4701-5p and transcription level HMGA1 were determined by PrimeScript RT Reagent Kit (Applied Biosystems, cat no. 4387424). Total RNA was collected by TRIzol reagent (Invitrogen, cat no. 12183555) and was used as template for one-step qRT-PCR detection. The RT-PCR master mix was prepared following the instruction and the experiment was conducted in Applied Biosystems™ 7500 Fast Real-Time PCR System. The controls used were GAPDH and U6 for mRNA or miRNA, respectively. All the primers were obtained from Sangon Biotech (Sangon, Shanghai, China).

### Dual luciferase reporter assay

The binding sites between miRNA-4701-5p and HMGA1 were predicated by bioinformatics website, and the HMGA1-3’UTR sequence with the binding site mutation was used as the control. The wild type and mutant sequences of human HMGA1-3’UTR were amplified and inserted downstream of its luciferase gene promoter (PGL3-HMGA1-3’UTR-wt or PGL3-HMGA1-3’UTR-mut). Then PGL3-HMGA1-3’UTR-wt, PGL3-HMGA1-3’UTR-mut, miRNA-4701-5p mimic or mimic control were transfected into HEK293T cells. After 48 h, the luciferase activity was analyzed by Dual-Luciferase® Reporter Assay System (Promega, Madison, WI, USA) in accordance with the manufacturer’s direction.

### Statistical analysis

Statistical analysis was carried out using SPSS 22.0 software. Comparisons between different groups were analyzed by Student's t-test and one-way analysis of variance (ANOVA). The data were displayed as the mean ± standard deviation from three independent experiments. If *P* < 0.05, the difference is defined as statistically significant.

## Results

### IL-1β treatment induces inflammation injury, decreased the expression of miRNA-4701-5p and enhanced HMGA1 expression in CHON-001 cells

**Fig. 1:**
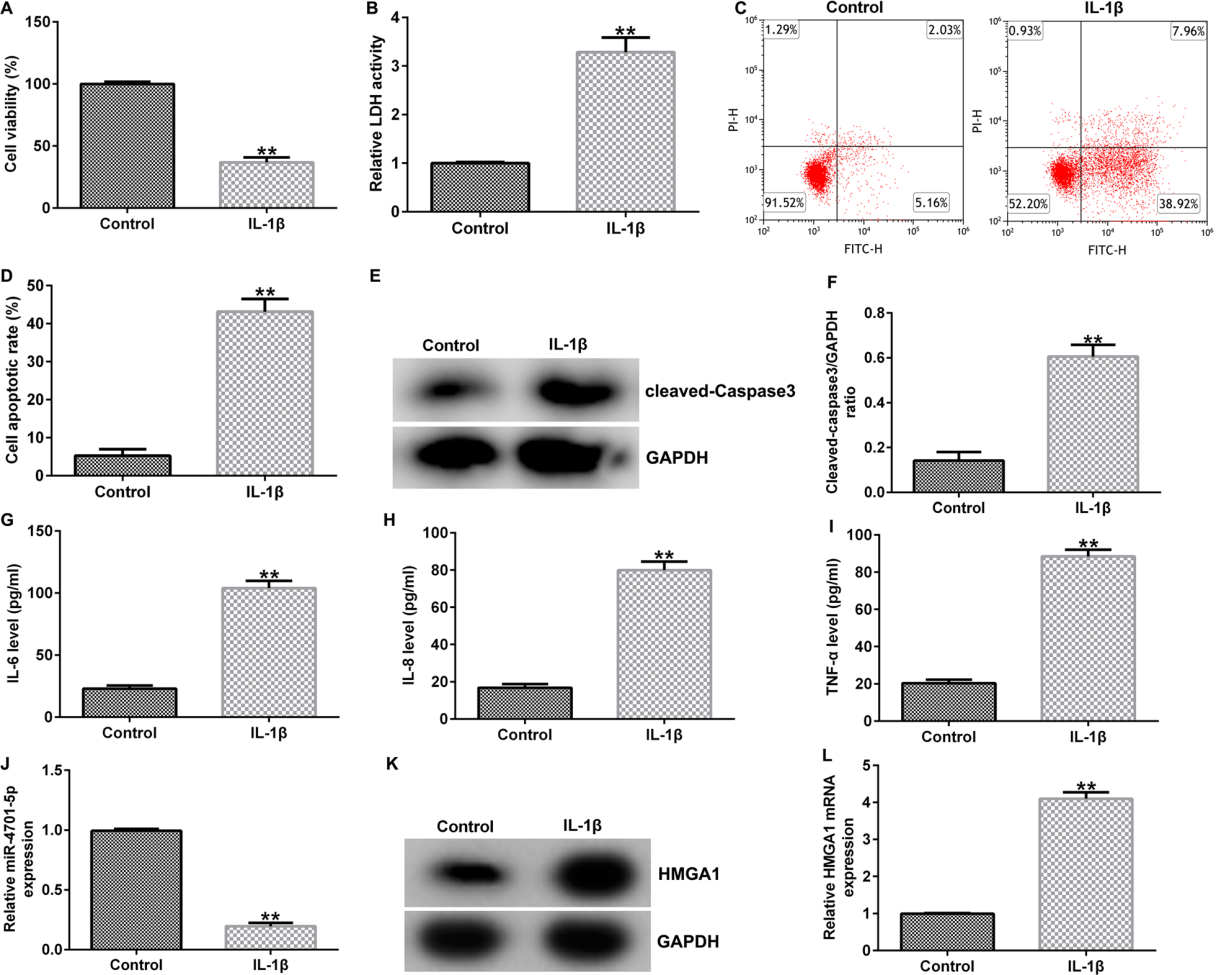
Expression of miRNA-4701-5p and HMGA1 in IL-1β induced CHON-001 cells. CHON-001 cells were exposed to 10 ng/ml IL-1β for 12 h. **A** CHON-001 cells proliferation was assessed by MTT assay. **B** The cytotoxicity of CHON-001 cells was accessed by LDH quantification. **C**, **D** Flow cytometry analysis of apoptotic cell. **E**, **F** Detection of cleaved-caspase3 expressions. **G**–**I** The secretion of IL-6, IL-8 and TNF-α in the supernatant of CHON-001 cells was determined by ELISA. **J** qRT-PCR analysis of miRNA-4701-5p level. **K**, **L** Western blot and qRT-PCR analysis of HMGA1 expression. **p < 0.01 versus control

CHON-001 cells were exposed to 10 ng/ml IL-1β for 12 h. Cell viability was determined by MTT assay and cytotoxicity was measured by LDH release. The results showed that IL-1β treatment led to decreased CHON-001 cells proliferation (Fig. 1A), and improved LDH release in CHON-001 cells (Fig. 1B). Apoptotic CHON-001 cells were analyzed using flow cytometry assay and cleaved-caspase3 level was determined using western blot assay. Results from Fig. 1C, D indicated that IL-1β treatment significantly enhanced the number of apoptotic CHON-001 cells, and obviously promoted cleaved-caspase3 expression and cleaved-caspase3/GAPDH ratio (Fig. 1E, F). Further experimental results demonstrated that IL-1β treatment could remarkably increase the secretion of IL-6, IL-8 and TNF-α (Fig. 1G–I). Analysis from qRT-PCR and western blot suggested that miRNA-4701-5p was down-regulated (Fig. 1J) and HMGA1 was up-regulated (Fig. 1K, L) in IL-1β-induced CHON-001 cells.

### miRNA-4701-5p binds directly with HMGA1

**Fig. 2:**
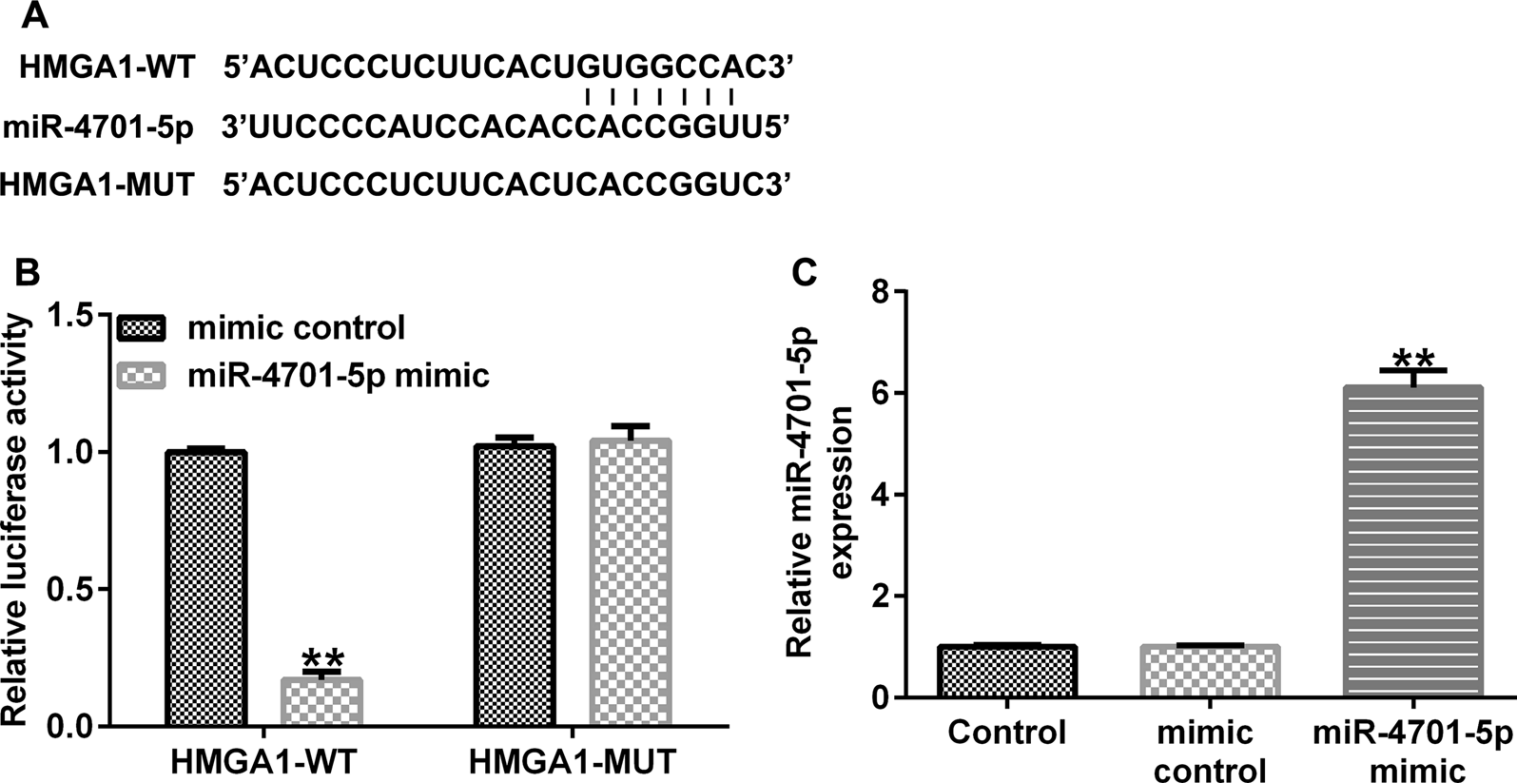
miRNA-4701-5p binds directly with HMGA1. **A** Bioinformatics software Starbase v2.0 analysis predicted the miRNA-4701-5p on HMGA1 3'UTR. **B** Dual luciferase reporter assay verified the relationship between miRNA-4701-5p and HMGA1. **C** The expression of miRNA-4701-5p in HEK293T cells was measured using qRT-PCR. **p < 0.01 versus mimic control

Bioinformatics software Starbase v2.0 analysis confirmed a potential miRNA-4701-5p binding site on HMGA1 3'-UTR (Fig. 2A), and the interaction was verified by dual luciferase reporter assay (Fig. 2B). The pGL-HMGA1-wt vector luciferase activity was declined after miRNA-4701-5p mimic co-transfection; however, there was no significant change in the luciferase activity of pGL-HMGA1-mut vector. And the expression of miRNA-4701-5p was significantly increased by miRNA-4701-5p mimic transfection in HEK293T cells (Fig. 2C).

### miRNA-4701-5p alleviates IL-1β-induced CHON-001 cells inflammatory injury by decreasing the expression of HMGA1

Based on previous research, we found that miRNA-4701-5p and HMGA1 were both dysregulated in CHON-001 cells and there was an interaction between miRNA-4701-5p and HMGA1. Therefore, we then explored whether HMGA1 could be regulated by miRNA-4701-5p in CHON-001 cells. Mimic control, miRNA-4701-5p mimic, control-plasmid or HMGA1-plasmid were transfected into CHON-001 cells for 24 h. The expression and transcription levels of HMGA1 were determined using western blot assay and qRT-PCR, respectively.

**Fig. 3:**
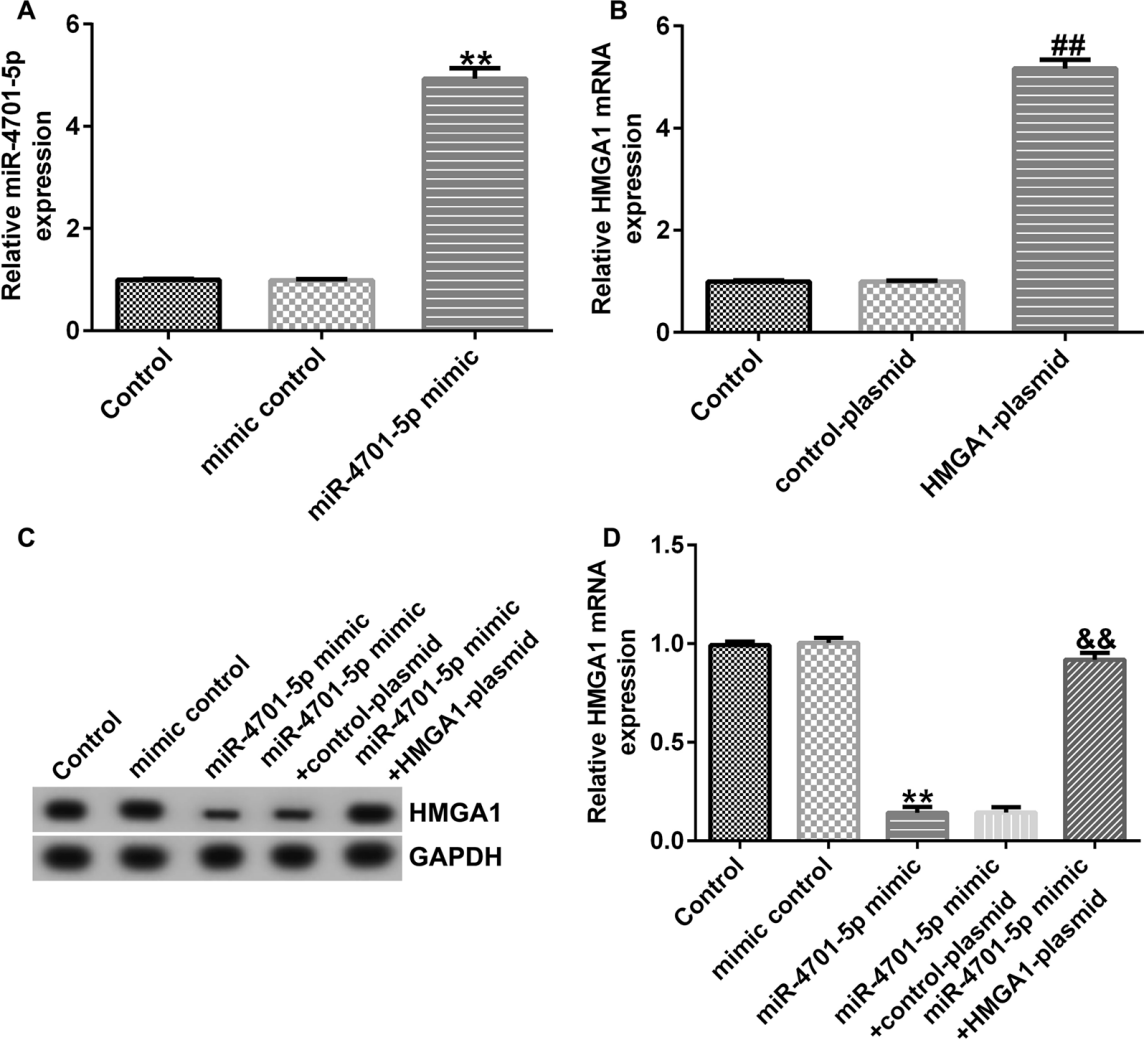
miRNA-4701-5p negatively regulates HMGA1 expression in CHON-001 cells. CHON-001 cells were transfected with mimic control, miRNA-4701-5p mimic, control-plasmid, HMGA1-plasmid for 24 h, respectively. The expression and transcription levels of HMGA1 were determined using western blot assay and qRT-PCR, respectively. **A** The expression of miRNA-4701-5p was determined by qRT-PCR. **B** The transcription levels of HMGA1 were determined by qRT-PCR, respectively. **C**, **D** The protein expression and transcription levels of HMGA1 were determined using western blot assay and qRT-PCR, respectively. **p < 0.01 versus mimic control; ^##^*p* < 0.01 versus control-plasmid; ^&&^*p* < 0.01 versus miRNA-4701-5p mimic + control-plasmid

miRNA-4701-5p mimic transfection markedly promoted miRNA-4701-5p expression (Fig. 3A), and the expression and transcription levels of HMGA1 were significantly increased by HMGA1-plasmid transfection (Fig. 3B). The expression and transcription levels of HMGA1 were significantly decreased in miRNA-4701-5p mimic transfected CHON-001 cells, and the decrease was obviously reversed by HMGA1-plasmid co-transfection (Fig. 3C, D), indicating that HMGA1 was negatively regulated by miRNA-4701-5p.

Then, we investigated the roles of miRNA-4701-5p/ HMGA1 regulation axis in OA in vitro model. CHON-001 cells were transfected with mimic control, miRNA-4701-5p mimic, control-plasmid or HMGA1-plasmid for 24 h, respectively, then the cells were cultured in 10 ng/ml IL-1β for 12 h.

**Fig. 4:**
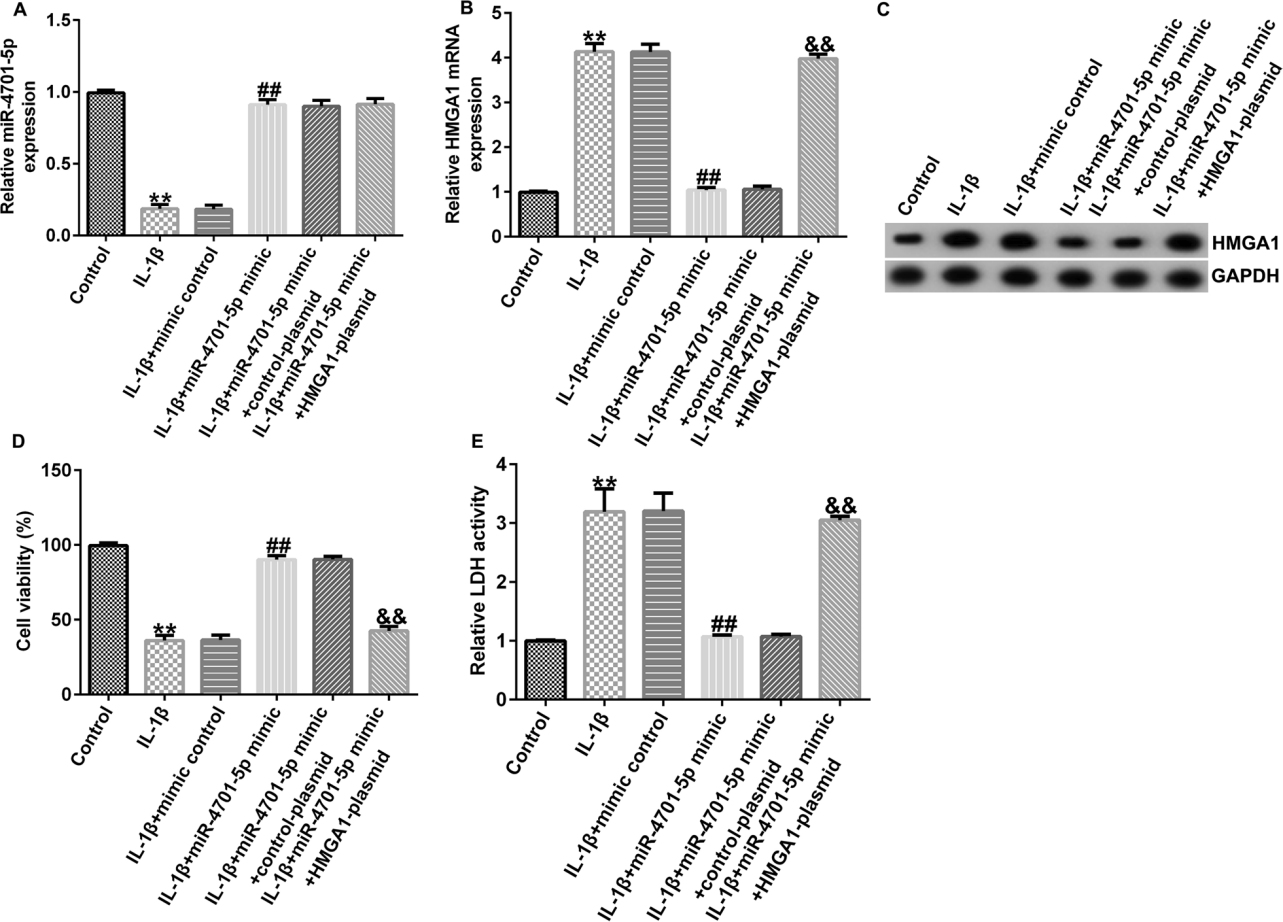
miRNA-4701-5p enhanced IL-1β-stimulated CHON-001 cells viability by down-regulating HMGA1. CHON-001 cells were transfected with mimic control, miRNA-4701-5p mimic, control-plasmid, HMGA1-plasmid for 24 h, respectively. Then the cells were induced by 10 ng/ml IL-1β for 12 h. **A** qRT-PCR analysis of miRNA-4701-5p expression. **B**, **C** Western blot assay and qRT-PCR analysis of HMGA1 expressions in different groups. **D** CHON-001 cells proliferation ability was detected by MTT assay. **E** The cytotoxicity of CHON-001 cells was accessed by LDH quantification. **p < 0.01 versus control; ^##^*p* < 0.01 versus IL-1β + mimic control; ^&&^*p* < 0.01 versus IL-1β + miRNA-4701-5p mimic + control-plasmid

MiRNA-4701-5p was down-expressed and HMGA1 was over-expressed in IL-1β stimulated CHON-001 cells. We also found that miRNA-4701-5p mimic transfection could significantly increase the expression of miRNA-4701-5p and the expression of HMGA1 was significantly decreased by miRNA-4701-5p mimic transfection. Moreover, after IL-1β treatment, the expression of miRNA-4701-5p had no distinct difference by miRNA-4701-5p mimic and HMGA1-plasmid co-transfection compared with miRNA-4701-5p mimic transfection, while the expression and transcription levels of HMGA1 were significantly improved by miRNA-4701-5p mimic and HMGA1-plasmid co-transfection compared with miRNA-4701-5p mimic transfection (Fig. 4A–C).

**Fig. 5:**
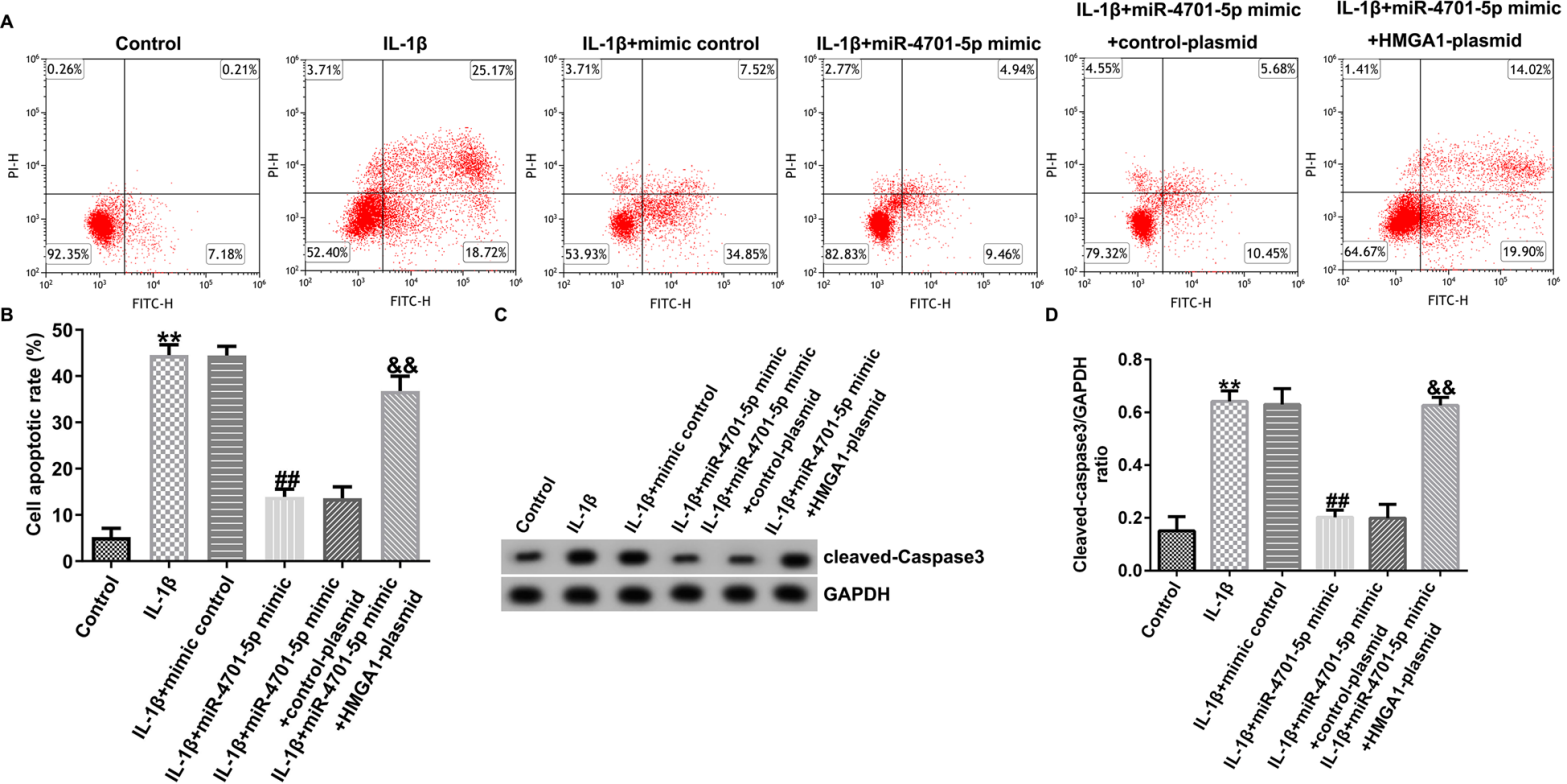
miRNA-4701-5p reduced the apoptosis of IL-1β induced CHON-001 cells by downregulating HMGA1. **A**, **B** Flow cytometry analysis of apoptotic cell. **C**, **D** Detection of cleaved-caspase3 expression and cleaved-caspase3/GAPDH ratio using western blot assay. **p < 0.01 versus control; ^##^*p* < 0.01 versus IL-1β + mimic control; ^&&^*p* < 0.01 versus IL-1β + miRNA-4701-5p mimic + control-plasmid

The proliferation ability and cytotoxicity of CHON-001 cells were assessed using MTT and LDH release assay, respectively. The results indicated that IL-1β treatment obviously decreased CHON-001 cells proliferation, while we observed the opposite results in miRNA-4701-5p mimic group, and the increase on proliferation induced by miRNA-4701-5p overexpression in IL-1β treated CHON-001 cells could be reversed by HMGA1 overexpression. Furthermore, the release of LDH in CHON-001 cells was markedly improved after IL-1β treatment, and miRNA-4701-5p overexpression obviously inhibited the release of LDH, while the inhibitory effect on LDH release caused by miRNA-4701-5p overexpression was obviously reversed by HMGA1 overexpression (Fig. 4D, E).

Cell apoptosis was determined using flow cytometry assay and cleaved-caspase3 expression was determined through western blot assay. We observed that CHON-001 cells apoptosis was markedly increased in IL-1β group, and miRNA-4701-5p overexpression markedly declined the apoptosis of IL-1β treated CHON-001 cells, while HMGA1 overexpression could reverse the apoptosis decline induced by miRNA-4701-5p overexpression (Fig. 5A, B). In addition, IL-1β treatment resulted in the promotion of cleaved-caspase3 expression and the ratio of cleaved-caspase3/GAPDH, while we observed the opposite results in miRNA-4701-5p overexpression group, and the decrease on the expression of cleaved-caspase3 and cleaved-caspase3/GAPDH ratio caused by miRNA-4701-5p overexpression were markedly reversed by HMGA1 overexpression (Fig. 5C, D).

**Fig. 6:**
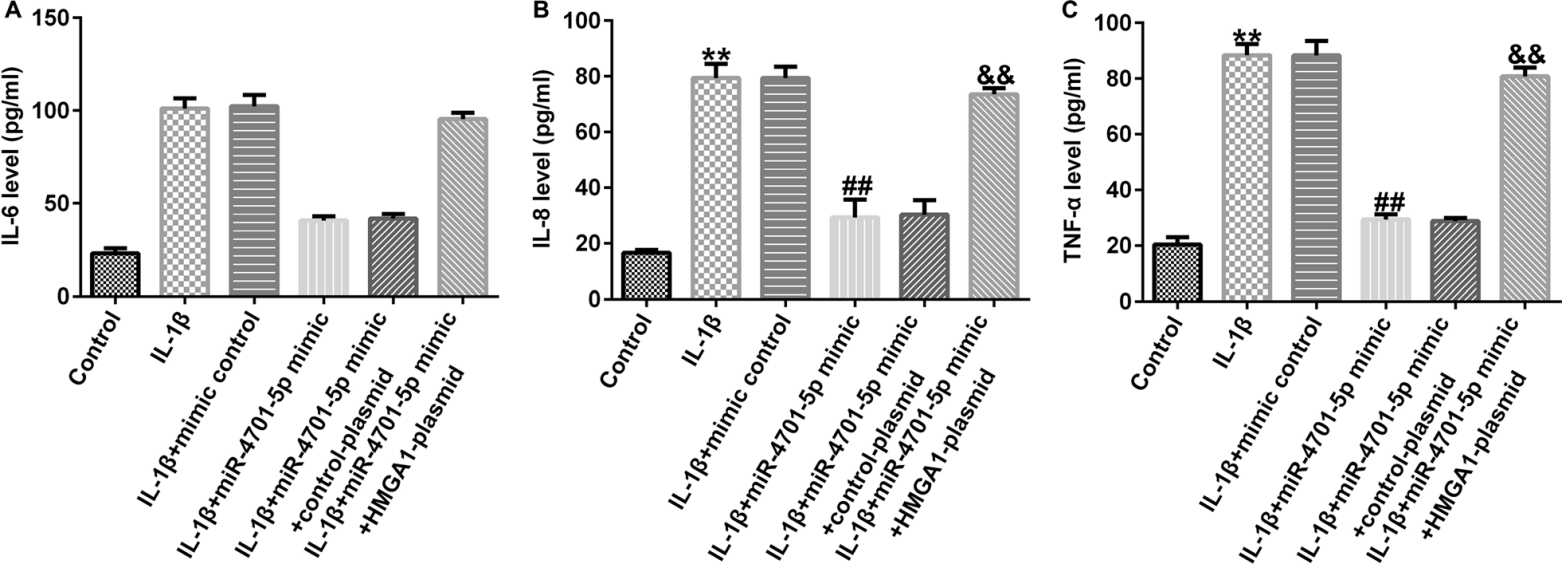
miRNA-4701-5p inhibited the inflammation of IL-1β- induced CHON-001 cells by downregulating HMGA1. The inflammation of CHON-001 cells was assessed using determining the secretion of IL-6 (**A**), IL-8 (**B**) and TNF-α (**C**) via ELISA assay. **p < 0.01 versus control; ^##^*p* < 0.01 versus IL-1β + mimic control; ^&&^*p* < 0.01 versus IL-1β + miRNA-4701-5p mimic + control-plasmid

The inflammation of CHON-001 cells was accessed by evaluating the release of IL-6, IL-8 and TNF-α. The experimental data suggested that IL-1β promoted the secretion of IL-6, IL-8 and TNF-α in treated CHON-001 cells, and the secretion of IL-6, IL-8 and TNF-α were markedly decreased by miRNA-4701-5p overexpression, while HMGA1-plasmid could abolish the increase of IL-6, IL-8 and TNF-α secretion (Fig. 6A–C).

## Discussion

OA is the most popular joint disease and the primary factor of disability, it is a chronic progressive joint disease [1]. The basic pathological changes of OA are characterized by cartilage degeneration and loss as well as bone regeneration at the joint margin and subchondral, mostly in the knee joint [3, 4, 31]. OA affects about 34% of Americans over the age of 65. As life expectancy increases, the incidence of OA has increased over the years [32]. Patients with OA suffering chronic intermittent joint pain, stiffness, swelling associated with joint dysfunction as the main performance, affecting the quality of life of patients [33].

In recent years, the role of miRNAs in musculoskeletal conditions have attracted the attention of scholars, and the role of miRNAs in OA has been extensively studied [34–36]. For example, Endisha et al. [37] reported that miRNA-34a-5p could promote joint destruction during osteoarthritis. Ding et al. [38] indicated that miRNA-93 inhibits chondrocyte apoptosis and inflammation in osteoarthritis by targeting the TLR4/NF-kappaB signaling pathway. Zhong et al. [17] suggested that miRNA-335-5p relieves chondrocyte inflammation by activating autophagy in osteoarthritis. In this study, we for the first investigated the role of miRNA-4701-5p in OA.

IL-1β is a vital pro-inflammatory factor and chondrocytes stimulated by IL-1β have been widely used as a model in OA research [39, 40]. Consistent with previous study [39], this study revealed that IL-1β stimulation significantly induced cytotoxicity and apoptosis of CHON-001cells, the inflammation of CHON-001cells was markedly increased by IL-1β stimulation. Thus, IL-1β stimulated chondrocytes were used as the in vitro model to study the role of miRNA-4701-5p in OA in vitro. Bioinformatics software analysis we found that miRNA-4701-5p contains hundreds of potential target genes including HMGA1. And the data of this study revealed that HMGA1 is a direct target of miRNA-4701-5p. Several studies have suggested that HMGA1 was highly expressed in cartilage from OA patients, and HMGA1 may be a causative factor in OA [29, 30]. In our report, miRNA-4701-5p was obviously down-regulated and HMGA1 was up-regulated in IL-1β stimulated CHON-001cells. The findings suggested that miRNA-4701-5p might be involved in OA through the regulation of HMGA1. We then explored whether miRNA-4701-5p affected IL-1β stimulated chondrocytes via targeting HMGA1.

The reduction of chondrocytes in the late stage of OA was accompanied by cavitation of the joint space, suggesting that cartilage cell death is a key feature of the progression of osteoarthritis [9, 41]. The proliferation of chondrocytes was significantly decreased and cytotoxicity of chondrocytes was significantly improved by IL-1β stimulation, and all these changes were significantly reversed by miRNA-4701-5p overexpression. Joint tissue inflammation and chondrocyte apoptosis have greatly contributed to the pathogenesis of OA [42, 43]. Meanwhile, the findings indicated that IL-1β induced cell apoptosis in chondrocytes was inhibited by miRNA-4701-5p up-regulation. Caspases are crucial mediators of programmed cell death (apoptosis). Among them, caspase-3 is a frequently activated death protease, catalyzing the specific cleavage of many key cellular proteins [7]. This study revealed that IL-1β enhanced cleaved-Caspase3 was significantly inhibited by miRNA-4701-5p. Moreover, we demonstrated that IL-1β stimulation could significantly induced inflammation of CHON-001cells, and miRNA-4701-5p overexpression could significantly relieve IL-1β-induced inflammation in CHON-001cells. It was worth mentioning that all the effects of miRNA-4701-5p overexpression on IL-1β-induced CHON-001cells could be reversed by HMGA1 overexpression.

There were also some limitations of current study. For example, this study did not study the expression of miRNA-4701-5p and HMGA1 in the cartilage tissue of OA patients. Besides, this study did not conduct in vivo experiments to verify the role of miRNA-4701-5p in OA. We will conduct these studies in the future.

In conclusion, our results for the first time revealed that miRNA-4701-5p and HMGA1 were dysregulated in IL-1β-stimulated CHON-001cells and HMGA1 was a target gene of miRNA-4701-5p. miRNA-4701-5p could significantly relieve IL-1β-induced injury on CHON-001cells by targeting HMGA1.

## Data Availability

The datasets used and/or analyzed during the current study are available from the corresponding author upon reasonable request.
