# Correction: microRNA-4701-5p protects against interleukin-1β induced human chondrocyte CHON-001 cells injury via modulating HMGA1

**DOI:** 10.1186/s13018-025-05731-1

**Published:** 2025-04-09

**Authors:** Hui Zhang, Cheng Chen, Jie Song

**Affiliations:** 1 Department of Orthopedics, Huangshi Central Hospital, Edong Healthcare Group, Affiliated Hospital of Hubei Polytechnic University, Huangshi, 435000 China; 2 Department of Geriatrics, Huangshi Central Hospital, Edong Healthcare Group, Affiliated Hospital of Hubei Polytechnic University, No. 141 Tianjin Road, Huangshi, 435000 China

**Correction: Journal of Orthopaedic Surgery and Research (2022) 17:246** 10.1186/s13018-022-03083-8

In this article, Fig. 5 appeared incorrectly and have now been corrected in the original publication. For completeness and transparency, the incorrect and correct versions of Fig. 5 are displayed below.

Incorrect Fig. 5
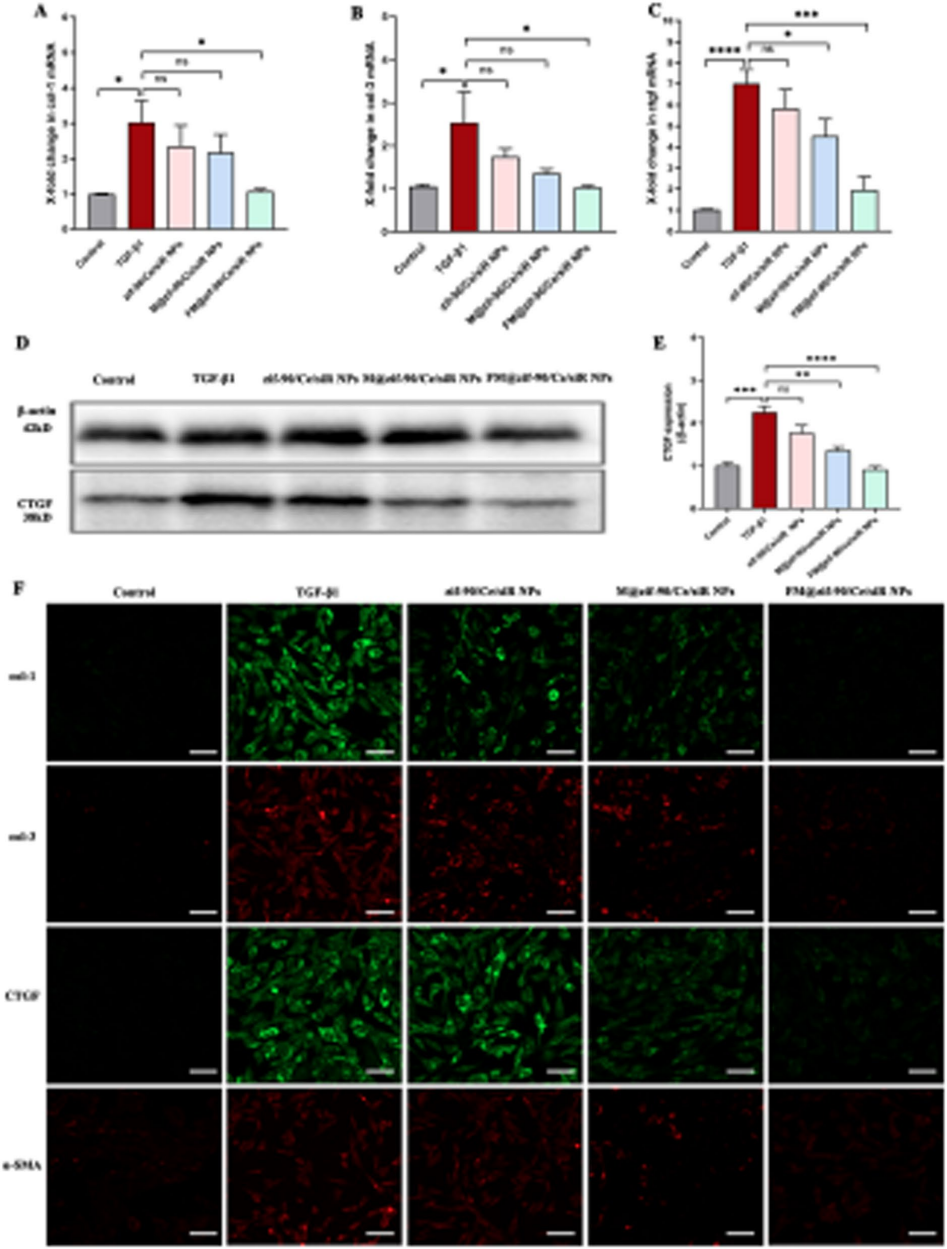


Correct Fig. 5
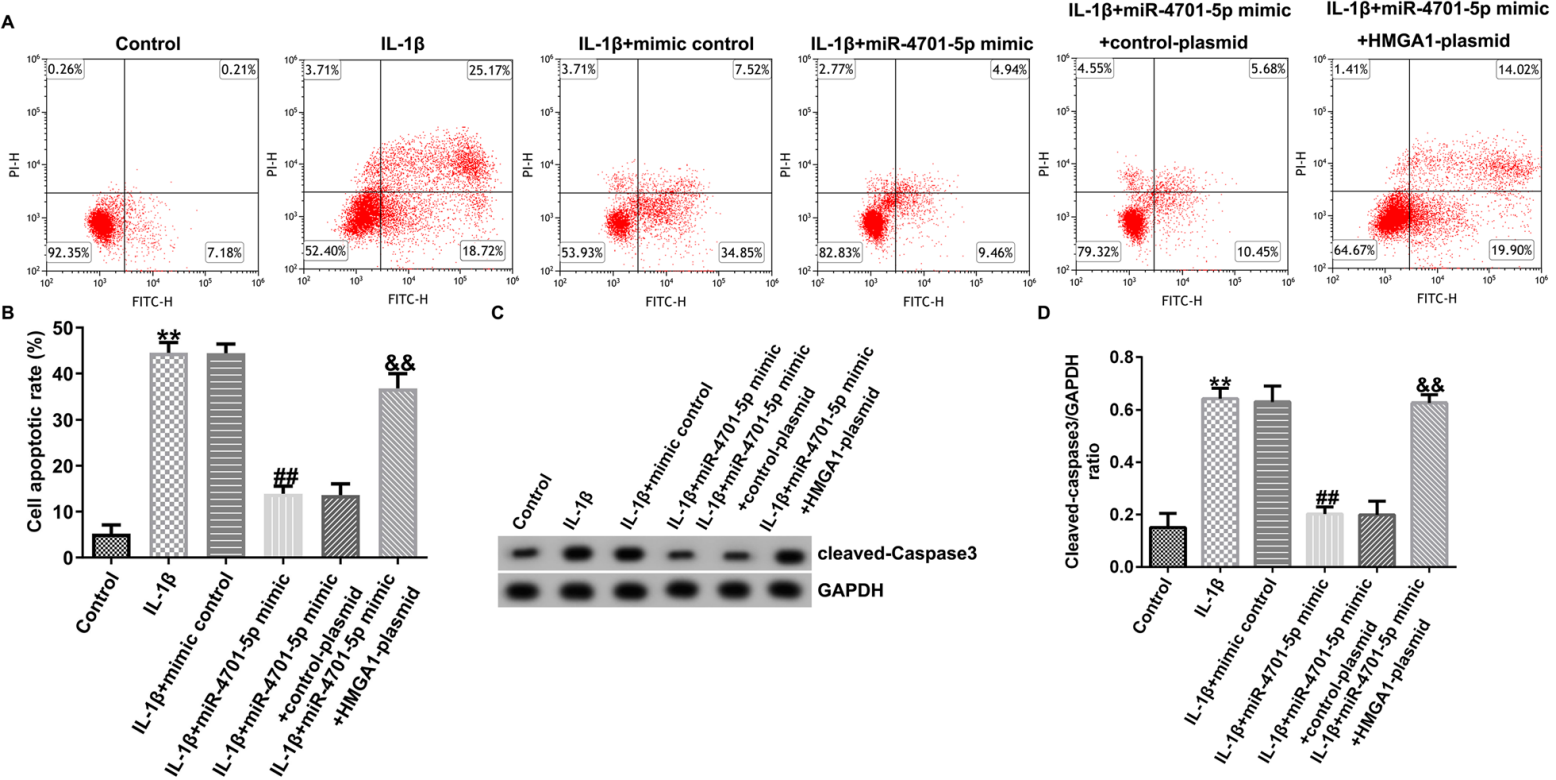


The original article has been corrected.

